# Danshen Formulae for Cancer: A Systematic Review and Meta-Analysis of High-Quality Randomized Controlled Trials

**DOI:** 10.1155/2019/2310639

**Published:** 2019-04-02

**Authors:** Tianqi Wang, Xianjun Fu, Zhenguo Wang

**Affiliations:** ^1^Traditional Chinese Medicine history and literature, Institute for Literature and Culture of Chinese Medicine, Shandong University of Traditional Chinese Medicine, Jinan 250355, China; ^2^Institute for Literature and Culture of Chinese Medicine, Shandong University of Traditional Chinese Medicine, 250355 Jinan, China

## Abstract

**Objective:**

Cancer is one of the most dangerous diseases to human life and there is no radical cure for it. In this paper, we compiled quantities of case history to evaluate the current available evidence of herbal Danshen (Radix Salviae Miltiorrhizae, RSM) formulae for the treatment of cancer by means of the high-quality randomized controlled trials (RCTs).

**Methods:**

English and Chinese electronic databases were searched from PubMed, the Cochrane Library, EMBASE, and the China National Knowledge Infrastructure (CNKI), VIP database, Wanfang database until September 2018. The methodological quality of the included studies was evaluated by using the method of Cocharne evidence-based medicine system evaluation, the quality was evaluated by screening the literature that met the requirements, and the Review Manager 5.3 was used for statistical analysis. The pooled odds ratio (OR) with 95% CIs was used to estimate the correlation between Danshen formulae and therapeutic effects.

**Results:**

Thirteen RCTs with 1045 participants were identified. The studies investigated the lung cancer (n = 5), leukemia (n = 3), liver cancer (n = 3), breast or colon cancer (n = 1), and gastric cancer (n = 1). A total of 83 traditional Chinese medicines were used in all prescriptions and there were 3 different dosage forms. Meta-analysis suggested that Danshen formulae had a significant effect on RR (response rate) (OR 2.38, 95% CI 1.66-3.42), 1-year survival (OR 1.70 95% CI 1.22-2.36), 3-year survival (OR 2.78, 95% CI 1.62-4.78), and 5-year survival (OR 8.45, 95% CI 2.53-28.27).

**Conclusion:**

The current research results showed that Danshen formulae combined with chemotherapy for cancer treatment was better than conventional drug treatment plan alone.

## 1. Introduction

Cancer, also known as malignant tumors, can destroy the structure and function of tissues and organs and cause necrotic hemorrhage and infection, and patients may eventually die due to organ failure. In recent years, the incidence of malignant tumors has increased. In the European Union, it is estimated that there are about 1.4 million new cancer cases in a year, including 1.2 million women, and about 70,000 men and 55,000 women die of cancer [1]. In the United States, cancer morbidity and mortality will continue to rise, and lung cancer is expected to remain the number one cancer killer [2].

As one of the world's five most intractable diseases, cancer is an incurable disease. If it is not detected and treated in time, it can also be transferred to all parts of the body for growth and reproduction and finally lead to body weight loss, weakness, anemia, loss of appetite fever, damage of viscera function, etc. [3]. The current cancer treatment is mainly surgery, chemotherapy, and radiotherapy, but both of these treatments cannot reduce the recurrence and metastasis after surgery. It is necessary to cooperate with other treatments after the operation [4]. The effects of radiotherapy and chemotherapy are obvious, but the obvious disadvantage is side effects. Cancer patients will be weak because of illness, and their constitution will be worse after chemotherapy and chemotherapy, which will lead to a decline in the quality of life and even make the body weaker and unable to withstand the next treatment [5].

Traditional Chinese medicine (TCM) treatment is a traditional treatment method in China. For cancer, combination of traditional Chinese medicine can promote the rehabilitation of patients and prevent postoperative tumor recurrence and metastasis. At the same time, traditional Chinese medicine can reduce side effects by radiotherapy and chemotherapy and improve the quality of life (QOL) of patients and even improve the survival rate [6, 7]. The Radix Salviae Miltiorrhizae (Danshen) originated from “Shen Nong's Herbal Classic” is a well-known TCM herb (China Pharmacopoeia Committee, 2005), and it has been used in clinical practice for over 2000 years. However, there was no consensus on the role of Danshen formulae in cancer treatment [8–20]. To scientifically validate the efficacy and safety of the Danshen formulae, the meta-analysis evaluated the value of Danshen formulae for the treatment of cancer based on high quality randomized controlled trials (RCTs).

## 2. Methods

This systematic review and meta-analysis are based on the Preferred Reporting Items for Systematic Reviews and Meta Analyses: PRISMA statement search strategy [21].

### 2.1. Search Strategy

We conducted literature retrieval through 5 database systems including PubMed, the Cochrane Library, EMBASE, and the China National Knowledge Infrastructure (CNKI), VIP database, Wanfang database. The retrieval deadline was September 2018. The main search terms of this paper included the following three parts: (Traditional Chinese Medicine OR Traditional Medicine, Chinese OR TCM OR Zhong Yi Xue OR Chinese Traditional Medicine OR Chinese Medicine, Traditional OR herbal medicine OR Medicine, Chinese Traditional) AND (Neoplasia OR Neoplasias OR Neoplasm OR Tumors OR Tumor OR Cancer OR Malignant Neoplasms OR Malignant Neoplasm OR Neoplasm, Malignant OR Neoplasms, Malignant OR Malignancy OR Malignancies OR Neoplasms). There was no restriction on the type of language. In addition, we used manual references to previously published system reviews to manually search for additional related research. The specific herb name “Danshen” has not been specifically searched to ensure that as many herbal formulae as possible were included.

### 2.2. Inclusion Criteria

(1) Type of participants: researches involving patients with any type of cancer

(2) Type of study: only RCTs that assessed the efficacy and safety of cancer treatment were eligible

(3) Type of intervention: Danshen must be included in the herbal formula used in the experimental group. There were no restrictions on the form of the drug (e.g., decoction, injection, pill, and capsule), dosage, frequency, or treatment time. Control group medications include placebo or conventional medication

(4) Types of results: the efficacy of cancer treatment was evaluated through OS (overall survival), duration of overall response, duration of stable disease, DFS (disease-free survival), PFS (progression-free survival), TTP (time to progression), TTF (time to failure), DCR (disease control rate), ORR (overall response rate), RR (response rate), CBR (clinical benefit rate), and ORR (objective response rate). Secondary outcome measures were quality of life (QOL) or side effects (such as fatigue, pain, infection/fever, anemia, diarrhea, nausea and vomiting, hair loss, and myelosuppression)

(5) RoB scored≥4 points

### 2.3. Exclusion Criteria

If the above conditions were not met, the study was excluded. In addition, the following documents were also excluded: (1) duplicate publications and (2) case series, review, animal studies, and pharmacological experiments, (3) other TCM therapies, such as acupuncture, massage, qigong, moxibustion, and Taiji were included in the study.

### 2.4. Study Selection

The two reviewers selected the study by screening the title and summary of the selected qualifying RCTs independently. Obtain and read the full text of studies that may meet predefined criteria. When the data were duplicated, only the most recent information is selected. The differences in the study choices were resolved by discussion with the latter corresponding author.

### 2.5. Quality Assessment

The methodological quality of the included studies was assessed using the risk of bias (RoB) tools, according to Cochran's Systematic Review Handbook on interventions [22]. Including the following seven contents: (A) random sequence generation (selection bias); (B) allocation concealment (selection bias); (C) blinding of participants and personnel (performance bias); (D) blinding of outcome assessment (detection bias); (E) incomplete outcome data (attrition bias); (F) selective reporting (reporting bias); (G) other bias.

### 2.6. Data Extraction

Two reviewers used predesigned standard data extraction forms to independently extract data from eligible trials. The excerpts were as follows: (1) the year of publication and the name of the first author, the language of publication, the type of cancer; (2) the characteristics of the participants, including the number, gender, age; (3) treatment information, including intervention management details, treatment process, and side effects; (4) measurement of results.

### 2.7. Danshen Formulae Composition

In each study, components of Danshen formula were documented, with a frequency analysis of the types of cancer it treated and common drugs combined with it.

### 2.8. Data Analysis

Dates from eligible researches were aggregated and a quantitative summary was generated by Review Manager 5.3. Egger's test was carried out by Stata 12.0.

### 2.9. Effect Size

Dichotomous data were reported as odds ratio (OR) with 95% confidence intervals (CI). The OR was deliberate significant at the* P *< 0.05 level when value 1 was not taken into the 95% CI. The purpose of this article is to explore the effectiveness of the Danshen formulae; therefore, we take the number of positive events as weights.

### 2.10. Heterogeneity

The statistical heterogeneity of the trials was assessed using Cochran* Q* tests and *I*^2^ statistics. If there is no absence of heterogeneity or moderate heterogeneity (*P* > 0.1, *I*^2^ < 50%) [23], a fixed effect model (FEM) will be used; otherwise, a random effects model (REM) will be applied.

### 2.11. Publication Bias

If a research project contained more than 10 studies, the funnel plot were used to test publication bias.

### 2.12. Sensitivity Analysis

Sensitivity analysis was performed to test the effect of a single study on the combined effect by removing the individual survey. If the estimated value of the point after deleting a study fell beyond the 95% CI of the total effect amount (or was significantly different from the combined effect amount), we considered the study in question to have exerted a great influence on the combined effect amount and that this study required further review.

## 3. Results

### 3.1. Description of Studies

**Figure 1:**
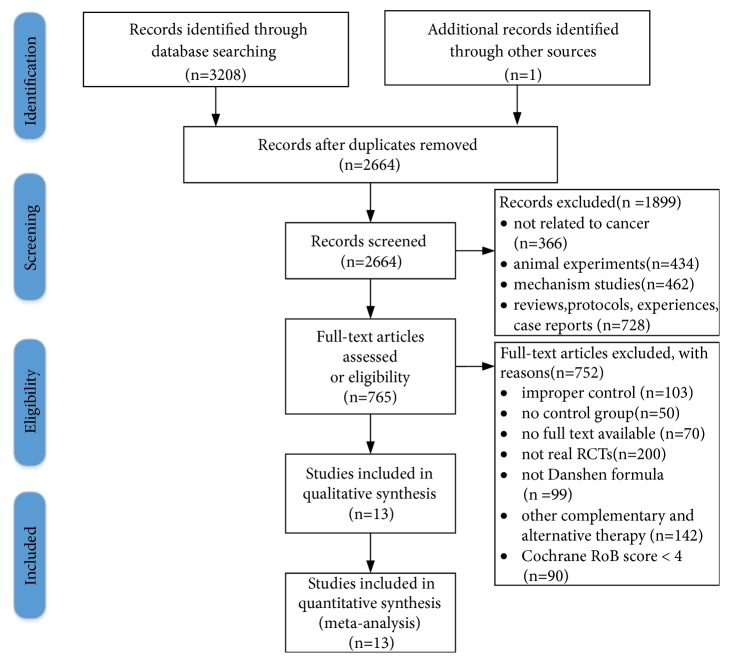
Flow diagram of literature search and selection.

A total of 3209 studies were searched by 4 electronic databases and other sources, and 2664 records were retained after deduplication. Of these, 366 studies were unrelated to cancer, 434 were animal experiments, 462 were mechanical experiments, and 728 were reviews, protocols, experiences, or case reports. By reading the full text, 762 studies, including 103 control interventions were inappropriate, lacking 50 of the control group and 70 items without full text, 200 items were not really RCTs, 99 items did not use the Danshen formula, and 142 included other CAM treatments, such as Acupuncture, Qigong, and 90 methodology that is of low quality. In the end, the study included 13 studies with 1045 patients that met the Cochrane Robb score of 4 and conducted a meta-analysis. A PRISMA flowed graph describe the search process and research options (Figure 1).

### 3.2. Basic Characters of the Included Studies

The characteristics of the 13 studies included the contents summarized in Table 1. All the eligible studies were conducted in China, with four articles published in English [9–11, 13] and the rest in Chinese [8, 12, 14–20]) (Table 1). In this study, there are 6 related lung cancers [9–11, 14, 19, 20], 3 related to Leukemia [12, 17, 18], 3 related to Liver cancer [8, 15, 19], one related to breast or colon cancer [13], and one related to gastric cancer [17] (Table 2). Because leukemia is a kind of malignant clonal disease of hematopoietic stem cells and belongs to the category of cancer, this study also included the research of leukemia. There were 10 RCTs with Overall response rate (RR) [8, 10–12, 14–19], 8 RCTs of them showed that Danshen formulae improved RR [10–14], and 2 RCTs suggested that Danshen formulae did not significantly improve RR [8, 11]. In 5 RCTs with survival rates, 4 RCTs of them indicated that Danshen Formulae improved survival rates [9, 15, 18, 20] and 1 RCT indicated that Danshen formulae did not significantly improve RR [8]. Seven RCTs were with side effects [10, 12–15, 17, 20] (Table 1). And the results showed 605 male patients and 440 female patients included in this study (Table 2). Among the 13 RCTs, 3 different Danshen formulae dosage forms were mentioned, 1 was a granule [17], 1 was a tablet [14], and the rest were decoction [8–13, 15, 16, 18–20]. In 8 Danshen formulae, Danshen plays a major role [8, 9, 12, 14–16, 19, 20] and improves the survival rate of patients; in 3 Danshen formulae, Danshen plays play an auxiliary role [10, 17, 18] and, in 2 RCTs, the real effect is that of other medicinal materials [11, 13]. It should be noted that one study did not involve radiotherapy, chemotherapy, or surgical treatment [19], while the rest were combined Chinese and western medicine (Table 3).

### 3.3. Description of the Danshen Formulae

Table 2 details the components of the Danshen formulae in each study. 83 kinds of herbs were used in 13 different Danshen formulae and three dosage forms were mentioned, namely, decoction (n=11), tablets (n=1), and formula particles (n=1) (Table 3). The 9 most commonly used herbs were Radix Salviae Miltiorrhizae (frequency=13), Astragalus mongholicus bunge (frequency=8), Rhizoma atractylodis Macrocephalae (frequency=5), Curcuma zedoaria (frequency=4), Agkistrodon seu bungarus (frequency=4), Poria (frequency=4), Adenophora stricta (frequency=3), Glycyrrhiza uralensis (frequency=3), and Portulaca grandiflora hook (frequency=3) (Table 4).

### 3.4. RoB Assessment

The RoB evaluation is shown in Table 4. All studies are described as random. Twelve RCTs mentioned random allocation methods, including random sampling, picking method, hospitalization time, completely randomized digital table, and stratified permuted block method, and the rest of the study had only the words “randomized grouping”. 1 RCT explicitly proposed that the study was conducted by a single blind method [9], 2 RCT explicitly proposed the use of double-blind method in the title or abstract [10, 13], and the rest of the studies were relatively vague about the blind method, and we need to get relevant information by reading the full text. As shown in the table, among the 13 studies, 3 articles scored 5 points [9, 10, 13], and the rest were scored 4 points [8, 11, 12, 14–20] (Table 5).

### 3.5. Effectiveness

#### 3.5.1. Cancer Patients Treated with Additional Danshen Formulae Have a Significantly High RR

**Figure 2:**
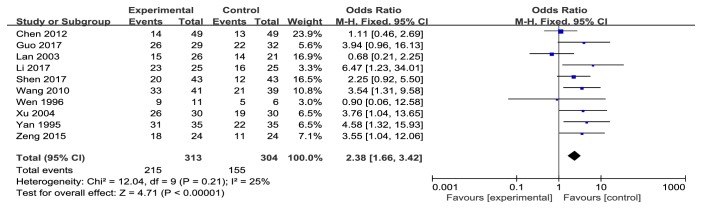
Meta-analysis of RR in experimental group and control group.

Ten studies [8, 10–12, 14–19] analyzed RR, indicating the RR of the experimental group was higher than that of the control group (OR 2.38, 95% CI 1.66-3.42) (Figure 2). Heterogeneity test* P* = 0.21,* I*^*2*^ = 25% showed 13 included articles with no heterogeneity, so the statistical analysis with fixed effects model. Pooled OR with 95% CIs showed* Z* = 4.71,* P*<0.00001 (Figure 2), suggesting that the difference was statistically significant. It can be considered that the RR of Danshen formulae with the general treatment regimen was higher to the control scheme without the Danshen formulae.

#### 3.5.2. 1-Year Survival Rate

**Figure 3:**
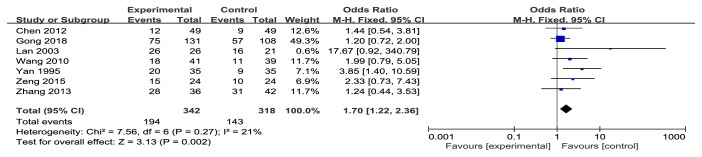
Meta-analysis of 1-year survival rate in experimental group and control group.

The total clinical efficacy rate 1-year survival rate between experimental group and control group was reported in 7 studies [8, 9, 11, 15, 18–20]. Compared with the control group, the 1-year survival rate was significantly improved after adding Danshen formulae (OR 1.70 95% CI 1.22-2.36,* Z* = 3.13,* P=*0.002), and there was a low heterogeneity (*P* = 0.27,* I*^*2*^ = 21%) (Figure 3).

#### 3.5.3. 3-Year Survival Rate

**Figure 4:**
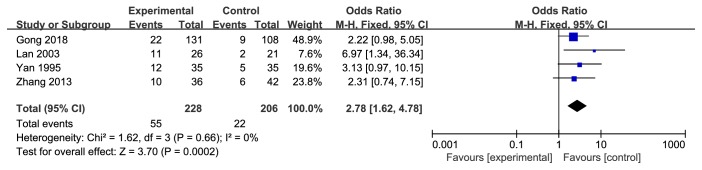
Meta-analysis of 3-year survival rate in experimental group and control group.

Four researches [9, 11, 18, 20] focused on 3-year survival rate between two groups. Pooled data showed that Danshen formulae were significantly better at increasing patient's 3-year survival (OR 2.78, 95% CI 1.62-4.78,* Z* = 3.70,* P=*0.0002) with no heterogeneity (*P* = 0.66,* I*^*2*^ = 0%) (Figure 4).

#### 3.5.4. 5-Year Survival Rate

**Figure 5:**
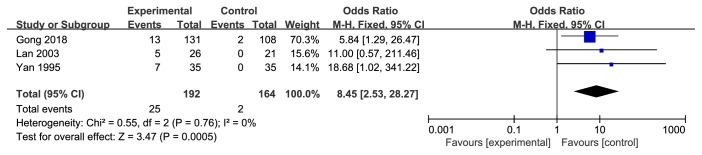
Meta-analysis of 5-year survival rate in experimental group and control group.

Three studies recorded 5-year survival rates [9, 11, 18]. Pooled data indicated that experimental group had a higher 5-year survival rate (OR 8.45, 95% CI 2.53-28.27,* Z* = 3.74,* P* = 0.0005) than control group with no heterogeneity (*P* = 0.76,* I*^*2*^ = 0%) (Figure 5).

### 3.6. Publication Bias

**Figure 6:**
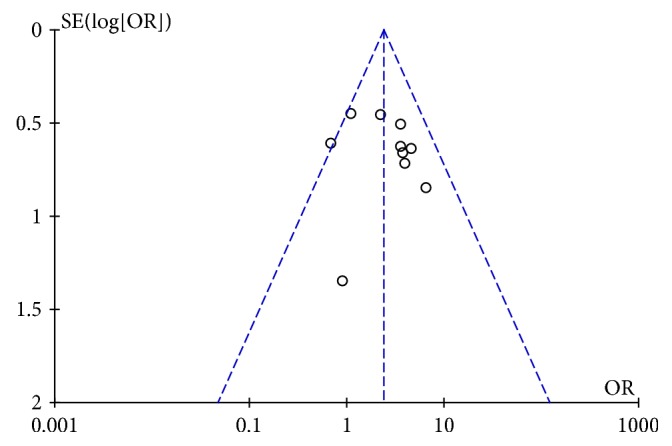
Funnel plot for RR analysis in experimental group and control group.

**Figure 7:**
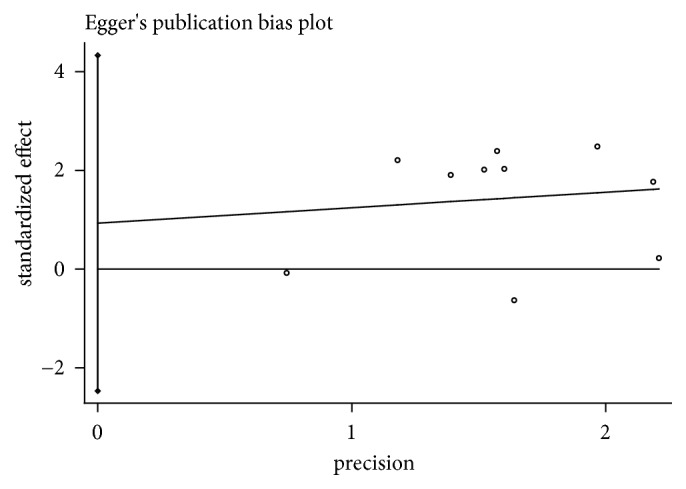
Egger's publication bias plot for RR analysis in experimental group and control group (*P*=1.07).

The funnel plot and further Egger's test were used to evaluate publication bias for RR of two groups of cancer patients. As the two results, though, showed a left-right asymmetry, but both* P*>0.05, suggesting that there was no publication bias (Figures 6 and 7).

### 3.7. Sensitivity Analysis

Our sensitivity analysis did not indicate that the results of any individual study would change the final outcome, indicating that none of the studies significantly affected the pooled OR and 95% CI.

### 3.8. Subgroup Analysis

**Figure 8:**
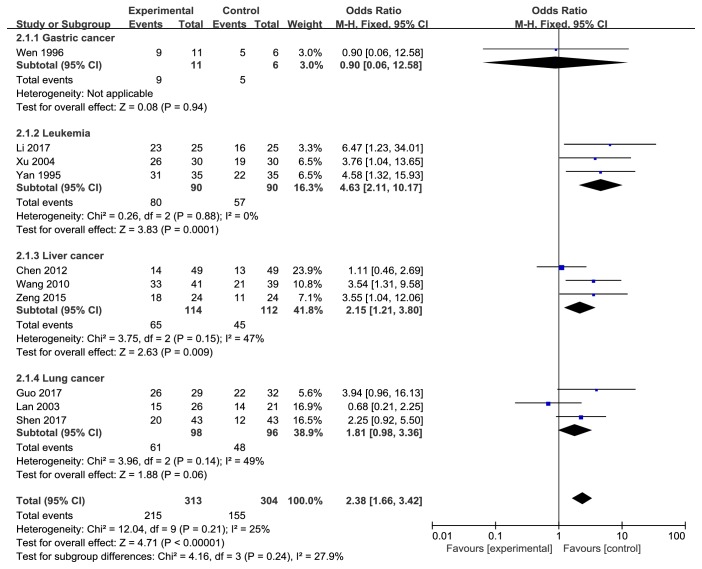
The RR analysis of Danshen formulae for different cancers.

To evaluate the effect of Danshen formulae for different cancers, we did a subgroup analysis. Danshen formulae did not show obvious beneficial effects in gastric cancer (OR 0.90 95% CI 0.06-12.58) and lung cancer (OR 1.81 95% CI 0.98-3.36), while it was good for treatment of leukemia (OR 4.63 95% CI 2.11-10.17) and liver cancer (OR 2.15 95% CI 1.21-3.80). Pooled data indicated that Danshen formulae had beneficial effects during the treatment progress in different cancers (OR 2.38 95% CI 1.66-3.42) (Figure 8).

## 4. Discussion

### 4.1. Summary of Evidence

In the past decades, much work has been reported in Chinese Herbal Medicine (HCM) in the treatment of cancer [8–20, 24, 25], and Zhang's review provided evidence for the effectiveness of Danshen in the treatment of cancer [26]. However, there has not been a meta-analysis to study the value of Danshen formulae in cancer treatment. This paper was a systematic review of 13 high-quality RCTs, including 1045 participants, to determine the efficacy and safety of the Danshen formulae for cancer treatment. Our study showed that the Danshen formulae provide statistically significant benefits in improving RR (OR 2.38 95% CI 1.66-3.42), 1-year survival (OR 1.70 95% CI 1.22-2.36), 3-year survival (OR 2.78, 95% CI 1.62-4.78), and 5-year survival rate (OR 8.45, 95% CI 2.53-28.27). Current evidence suggests that Danshen formulae can be used as an effective adjuvant for treat cancer.

### 4.2. Implications for Practice

Modern pharmacological studies were performed on more than 10 tanshinone monomers, including tanshinone I (TNI), tanshinone A (TNIIA), tanshinone B, and cryptotanshinone (CPT), from Danshen root. Tanshinone TNI are the main bioactive components, TNIIA, and implicit tanshinone (CPT); TNIIA activity in salvia miltiorrhiza is the strongest diterpene quinine pigment; TNI and CPT are effective cytotoxic agent and can induce apoptosis and the stagnation of the cell cycle; potential mechanisms involved include raised to promote apoptosis proteins such as p53, Bax, and p21 and inhibit antiapoptotic proteins, including the Bcl-2, survivin, and c-Myc and activated caspase protein to trigger apoptosis, by activating AMP activated protein kinase and extracellular signal regulating kinase (ERK) and suppress the target of pakamycin and 70 kDa ribosomal protein S6 kinase signaling pathways; TNIIA induces autophagic cell death in various cancer cells. Furthermore, TNIIA and TNI can inhibit the migration, invasion, and metastasis of cancer cells by changing the tissue inhibitors of matrix metalloproteinase and/or metalloproteinase [26, 27]. In addition, TNIIA can also promote the differentiation of several cancer cell types and regulate the CCAAT/enhancer binding protein (C/EBP)*β* and C/EBP homologous protein. Besides, in animal models, the side effects of TNIIA, TNI, and CPT were minimal [28].

In addition, Danshen root also has anti-inflammatory effects. TNIIA inhibits the NF-kB induced kinase/IkappaB alpha kinase (NIK/IKKalpha), while ERK1 suppress NF-*κ*B induced by LPS and c-Jun n-terminal kinase (JNK) pathway. The anti-inflammatory effects of TNIIA may be related to the inhibition of the Toll-like receptor (TLR) signaling pathway by TNF receptor-associated factor (TRAF) 2/3/6. TNI significantly inhibits the activity of IIA secreting phospholipase A2 (GIIA), thereby blocking the formation of prostaglandin E2 (PGE_2_) in LPS-activated macrophages [27, 29]. TNI and CPT also significantly inhibit IL-12 production in LPS-activated macrophages and interferon-*γ* production in lymphocytes. Recent studies have shown that Salvia miltiorrhiza extract inhibits the production of iNOS and COX-2 by regulating NF-*κ*B and MAPKs, thereby inhibiting the secretion of inflammatory cytokines. In LPS induced RAW264.7 macrophages, salvia miltiorrhiza extract reduced the secretion of nitric oxide (NO), tumor necrosis factor- (TNF-) *α*, and interleukin 6 (IL-6) and decreased the expression of inducible nitric oxide synthase (iNOS), cyclooxygenase-2 (COX-2), and NF-*κ*B. Moreover, salvia miltiorrhiza extract can significantly inhibit the activation of JNK1/2 and ERK1/2 induced by LPS and disrupt the TLR4 dimerization in LPS-induced RAW264.7 macrophages [29]. Therefore, the anti-inflammatory effect of Danshen root is partly due to the blocking of TLR4 dimerization, which can be used in clinical treatment of liver injury and infection during the anticancer strategies.

Therefore, Chinese medicine practitioners usually use traditional Chinese medicines similar to Danshen root, such as Scutellaria barbata and Hedyotis diffusa in the treatment of cancer [8–20]. It should be mentioned that the current meta-analysis is the first systematic review of the application of Danshen formulae in cancer-assisted treatment. The current meta-analysis found that Danshen formulae can improve the clinical efficiency in cancer treatment. After the addition of Danshen formulae, RR and survival rates were significantly improved. However, it is not clear which components of Danshen formulae have anticancer effects during the treatment, and what role Danshen root plays, which should be the goal of further research.

### 4.3. Limitations

All literatures for this meta-analysis were from China, among which 4 were in English and the rest were in Chinese, and only one was multicenter study [10]. Blind methods have been described vaguely in many studies and most of the references were scored 4 points. Moreover, all types of researches were single-center studies with a small sample size and lack of data support for multicenter, large RCTs. The funnel plot analysis also found obvious asymmetry between the left and the right of funnel plot, therefore, the effect of Danshen formulae in assisting cancer treatment may be exaggerated. In addition to all of the above, the cycle of cancer treatment is extremely long and an army of patients died as their condition worsens during the treatment process, which leads to follow-up work is difficult, resulting in the inability to obtain valid data for many studies, and some literature related to this study cannot be included. Therefore, to better explore the contribution of Danshen formulae in cancer treatment, more large-scale and higher standard studies are needed.

### 4.4. Implications for Further Studies

Nearly 50% of the RCTs included in our study are related to lung cancer, indicating that Danshen formulae may be more widely used in lung cancer. Lung cancer is one of the most dangerous diseases, and it has a variety of treatment options, but the death rate is stubbornly high [2]. Except lung cancer, the included studies also cover leukemia, liver cancer, breast, colon cancer, and gastric cancer, and our study found that 9 kinds of herbs were used in combination with Danshen root in cancer treatment, suggesting that the pharmacological effects of these drugs together may be a mechanism to improve clinical efficacy and reduce side effects. Therefore, this study provides the basis for the clinical treatment and scientific research of Danshen for cancer. In terms of gender ratio, we found that men have a higher risk of cancer than women (605/440), suggesting that men should pour more attention into prevention of cancer, which was also a limitation of this paper, indicating that we should avoid gender selection bias by recruiting women to a certain extent in future studies. By inputting the dose form of Danshen formulae to statistical analysis, we found that there were 1 used granule preparation, 1 used tablet, and the other 11 used decoctions. Ling proved that TCM preparations more safer, effective, and easier to use than decoctions of traditional Chinese medicine [30], and the present study showed that the clinical curative effect and dosage forms of CHM were interconnected, interdependent, and mutually reinforcing with each other; drug application shall be familiar with drug characteristics on the premise of fully considering disease characteristics and age constitution and patient and choose the appropriate dosage forms, through the appropriate method to give full play to the effect, and make the drug in patients with optimal clinical curative effect [31, 32]. Therefore, rational selection of drug dosage forms is beneficial to enlighten and create new drugs, which can better promote the further development and research of new dosage forms with higher drug absorption rate. In terms of the treatment of cancer, in traditional western medicine, chemotherapy and radiotherapy are the main treatments for cancer. The purpose of these therapies is to kill or destroy cancer cells. Unfortunately, for most cancer treatments, it is difficult to distinguish between cancer cells and normal healthy cells, which leads to damage to normal cells [33, 34]. The results of this injury are known as complications and side effects of cancer treatment. There were 10 included studies showed that the formulae of Danshen had significant effect in reducing the side effect of vomiting and blood toxicity, which suggested that we could cooperate with the Danshen formulae in the treatment of cancer in the future to reduce the gastrointestinal reaction of patients. The results of subgroup analysis suggested that Danshen formulae might be taken advantage of for cancer treatment. At last, the exact pathologic and clinical pharmacological mechanisms of cancer are still largely unknown and should be studied further.

## 5. Conclusion

Current findings suggested that Danshen formulae offered statistically significant benefits for cancer, which we generally considered safe. Thus, evidence from the existing study supported the use of Danshen formulae as a treatment for cancer. However, this study was based on several small‐sample studies. Therefore, studies with rigorous, large‐scale RCTs of Danshen formulae in treating of cancer were needed to further confirm its efficacy.

## Figures and Tables

**Table 1:** Basic characteristics of the included studies.

Included trial	Publication language	Study design and investi- gational sites	Type of cancer	No.of participants (male/female;age years)		Intervention		Outcome index	Intergroup difference
				Experimental	Control	Experimental	Control		
Chen 2012 [8]	Chinese	RCT,Single cencer;China	Primary Liver Cancer	38/11;44.7	41/8;47.1	Danshen formulae	Vinorelbine +Cisplatin(NP)+Placebo (Huangqi, Huangjing, Jiaogulan, Lingzhi, Cangzhu, Nvzhenzi, Huanglian et al.)	1.RR; 2.Survival rate;3.Improvement of symptoms	1,2.*P*>0.05; 3<0.05
Gong 2018 [9]	English	RCT,Single cencer;China	Non-small cell lung cancer (NSCLC)	80/51;40-78	66/42;41-80	Vinorelbine +Cisplatin(NP)+Danshen formulae	Platinum-based therapy +Placebo (Jiegeng, Xingren, Yuxingcao, Ziwan, Kuandong, Zhebeimu, Banxia, Hexiancao, Shengdiyu, Huangqin, Dongguazi, Chenpi, Zhuru et al.)	1.Survival Rates;2.MST	1,2.*P*<0.05
Guo 2017 [10]	English	RCT, Multi-cente;China	NSCLC	20/9;38-77	28/4;38-77	Platinum-based therapy +Danshen formulae	Radiotherapy	1.RR;2.Nausea; 3.Vomit;4.QOL; 5.Mildgastric/abdominal heaviness	1,2,3,5.*P*<0.05;4.P>0.05
Lan 2003 [11]	English	RCT,Single cencer;China	Lung cancer	16/10;70-77	13/8;70-79	Radiotherapy+Danshen formulae	DAP	RR(1.Short-term effect;2. Long-term effect)	1*.P*>0.052.*P*<0.01
Li 2017 [12]	Chinese	RCT,Single cencer;China	Leukemia	10/15;(35.42±15.13	13/12;37.97±13.45	DAP+Jiedu Huayu Fang	Radiotherapy+placebo (Xuanshen,Yiyiren, Chenqianzi, Yinchen, Difuzi, Chuanwu, Ganjiang, Rougui, Wuzhuyu, Xiangfuzi, Chenpi, Shanzha, Laifuzi, Sanqi, Ruxiang, Yujin et al.)	1.RR(CR+PR);2.Leukopenia; 3.Platelet(PLT)decreased; 4.Hemoglobin(HGB)decreased	1,3,4.*P*<0.05;2.*P*>0.05
Mok 2007 [13]	English	RCT,Single cencer;China	Breast or colon cancer	5/50;32-75	6/50;39-72	Radiotherapy+Danshen formulae	Paclitaxel Injection+Cisplatin Injection	1.HGB decreas -ed;2.Leukopenia;3.Neutropenia; 4.Thrombocytopenia;5.Nausea; 6.QOL	1-4,*P*>0.05;5,6.*P*<0.05
Shen 2017 [14]	Chinese	RCT,Single cencer;China	NSCLC	22/21;57.2 ±7.3	23/20;56.8±7.5	Paclitaxel Injection+Cisplatin Injection+Zilongjin tablet	3-DCRT	1.RR;2.Leucopenia;3.Erythropenia; 4.Thrombocytopenia;5.Nausea;6.DCR	1,2,3,5,6.*P*<0.05;4.*P*>0.05
Wang 2010 [15]	Chinese	RCT,Single cencer;China	Primary liver cancer	28/13;32-68	25/14;33-67	3-DCRT+Aixiao Aixiao Ruanganjian	UFTM/FMV	1.RR;2.Leucopenia;3.Platelet descend; 4.Hemoglobin reduction.5.T-cells; 6.Survival rate;7.Nausea	1-7.P<0.05
Wen 1996 [16]	Chinese	RCT,Single cencer;China	Gastric cancer	8/3;47-73	5/3;50-69	UFTM/FMV+Jianpi Kangai mixtures	DA/HA/IA	1.RR	1.*P*<0.05
Xu 2004 [17]	Chinese	RCT,Single cencer;China	Acute myleoid leukemia	19/11;11-65	20/10;12-64	DA/HA/IA+Danshen formulae	Radiotherapy+Placebo (TizisheN, huangbai, Baizhu, Gouqi, Huang jing, Tiandong, Maidong, Xuan shen, Nvzhenzi, Hanliancao, Pugonging, Banzhilian, Baihuasheshecao, Xiaoji et al.)	1.RR;2.Aminotransferase	1,2.*P*<0.05
Yan 1995 [18]	Chinese	RCT,Single cencer;China	Acute leukemia	16/19;14-60	18/17;17-60	Radiotherapy+Danshen formulae	General symptomatic treatment+Fufang Banmao Capsule	1.RR;2.Survival rate	1.*P*<0.05;2.*P*<0.01
Zeng 2015 [19]	Chinese	RCT,Single cencer;China	Liver cancer	20/4;52.96±7.14	19/5;53.95±10.25	General symptomatic treatment+Fuzhengkangai decoction	DDP+VP-16	1.RR;2.ALT;3.AST;4.GGT;5.AFP;6.CR+PR; 7. CR+PR+NC; 8.Survival rates; 9.Nausea;10.Vomiting	1,4,7.*P*<0.05; 2,3.P<; 5,6,8,9,10.*P*>0.05
Zhang 2013 [20]	Chinese	RCT,Single cencer;China	NSCLC	Total:48/30;55±2.1		DDP+VP-16d123+Xidan Tang	Vinorelbine +Cisplatin(NP)+Placebo (Huangqi, Huangjing, Jiaogulan, Lingzhi, Cangzhu, Nvzhenzi, Huanglian et al.)	1.Survival rate;2.Nausea, vomiting and loss of appetite	1.*P*<0.05; 2.*P*>0.05

**Table 2:** Types of cancer.

Types of cancer	Included trial(s)	Sex(male/female); Total	Frequency
Lung cancer	Gong 2018	146/93	5
	Guo 2017	48/13	
	Lan 2003	29/18	
	Shen 2017	45/41	
	Zhang 2013	48/30	
		Total:316/195	
Leukemia	Li 2017	23/27	3
	Xu 2004	39/21	
	Yan 1995	34/36	
		Total:96/84	
Liver cancer	Chen 2012	79/19	3
	Wang 2010	53/27	
	Zeng 2015	39/9	
		Total:171/55	
Gastric cancer	Wen 1996	11/6	1
		Total:11/6	
Breast or colon cancer	Mok 2007	11/100	1
		Total:11/100	

**Table 3:** The constituent of Danshen formulae in each included study.

Included trials	Chuanxiong formula	Ingredient			
		Latin name	English name	Chinese name	Dosage (g)
Chen 2012	Gexiazhuyu decotion	Radix salviae miltiorrhizae	Danshen root	Danshen	9
		Astragalus mongholicus	Milkvetch Root	Huangqi	14
		Semen persicae	Peach seed	Taoren	9
		Flos carthami	Safflowe	Honghua	6
		Paeoni	paeonol	Danpi	12
		Rhizoma cyperi	Nutgrass galingale rhizome	Xiangfu	10
		Fructus citri aurantii immaturus	Immature bitter orange	Zhiqiao	12
		Agkistrodon seu bungarus	Hedyotis diffusa	Baihuasheshecao	16
		Radix scrophulariae ningpoensis	Radix scrophulariae	Xuanshen	14

Gong 2018	Jupi Zhuru decoction	Radix salviae miltiorrhizae	Danshen root	Danshen	15
		Pericarpium citri reticulatae	Dried Tangerine	Chenpi	9
		Pinellia ternata	Rhizoma pinelliae	Jiangbanxia	9
		Bambusa turdoides munro	Bamboo shavings	Jiangzhuru	9
		Zizyphus jujuba	Chinese date	Dazao	9
		Glycyrrhiza uralensis	Liquorice root	Gancao	6

Guo 2017	TCM formulae decoction	Astragalus mongholicus	Milkvetch Root	Huangqi	30
		Rhizoma atractylodis macrocephalae	Largehead atractylodes	Baizhu	9
		Poria	Poria cocos	Fuling	15
		Radix glehniae	Coastal glehnia root	Beishashen	30
		Radix adenophorae	Fourleaf ladybell root	Nanshashen	30
		Radix asparagi	Cochinchinese asparagus root	Tiandong	15
		Radix ophiopogonIs	Dwarf lilyturf tuber	Maidong	15
		Radix salviae miltiorrhizae	Danshen root	Danshen	30
		Selaginella doederleinii hicr	Selaginella doederleinii	Shishangbai	30

Lan 2003	TCM formulae decoction	Adenophora stricta	Radix Adenophorae	Shashen	30
		Bulbus fritillariae cirrhosae	Tendrilleaf fritillary bulb	Chuanbeimu	10
		Astragalus mongholicus bunge	Milkvetch root	Huangqi	30
		Ophiopogon japonicus	Dwarf lilyturf tuber	Maidong	15
		Fallopia multiflora	Tuber fleeceflower root	Heshouwu	30
		Rehmannia glutinosa	Prepared rehmannia root	Shudihuang	10
		Dioscorea opposita	Common Yam Rhizome	Shanyao	30
		Alismatis Rhizoma	Alisma orientale	Zexie	15
		Fructus Corni	Cornus officinalis	Shanzhuyu	10
		Glycyrrhiza uralensis	Liquorice root	Gancao	10
		Rhizoma Phragmitis	Reed rhizome	Lugen	30
		Lonicera japonica Thunb	Lonicera japonica	Jinyinhua	15
		Morus alba L.	White mulberry root-bark	Sangbaipi	15
		Radix Sophorae Tonkinensis	Radix sophorae tonkinensis	Shandougen	15
		Gypsum fibrosum	Gypsum fibrosum	Shengshigao	30
		Scutellariae baicalensis	Radix scutellariae	Huangqin	15
		Codonopsis pilosula	Danshen root	Danshen	30
		Radix et rhizoma rhei	Rhubar	Dahuang	15

Li 2017	Jiedu Huayu Fang	Lygodium japonicum	Indigo Naturals	Qingdai	Not mentioned
		Paris polyphylla Smith	Flea	Zaoxiu	
		Fructus Psoraleae	Psoraleae	Buguzhi	
		Rhizoma Polygoni Cuspidati	Polygonum cuspidatum	Huzhang	
		Radix salviae miltiorrhizae	Danshen root	Danshen	
		Atractylodes macrocephala Koidz	Largehead atractylodes rhizo	Baizhu	
		Asarum sagittarioides	Cremastra appendiculata	Shancigu	
		Rhizoma Ligustici Chuanxiong	Sichuan lovage rhizome	Chuanxiong	

Mok 2007	TCM formulae granules	Curcuma zedoaria	Rhizoma curcumae	Ezhu	Not mentioned
		Sparganium simplex Huds	Rhizoma spargani	Sanleng	
		Radix salviae miltiorrhizae	Danshen root	Danshen	
		Leonurus artemisia	Herba leonuri	Yimucao	
		Radix scrophulariae	Scrophularia ningpoensis	Xuanshen	
		Polyporus	Polyporus umbellatus	Zhuling	
		Coix lacrymajobi	Coix seed	Yiyiren	
		Semen Plantaginis	Plantain seed	Cheqianzi	
		Herba lysimachiae	Longhairy antenoron Herb	Jinqiancao	
		Spora lygodii	Lygodium japonicum	Haijinsha	

Shen 2017	Zilongjin Tablet	Astragalus mongholicus	Milkvetch root	Huangqi	0.65g per piece
		Radix angelicae hinensis	Chinese angelica	Danggui	
		Solanum lyratum thunb	Solanum lyratum thunb	Baiying	
		Solanum nigrum	Nightshade	Longkui	
		Radixsalviae miltiorrhizae	Danshen root	Danshen	
		Portulaca grandiflora hook	Scutellariae barbatae	Banzhilian	
		Duchesnea indica	Duchesnea	Shemei	
		Turmeric Curcumae	Turmeric	Yujin	

Wang 2010	Aixiao Ruanganjian	Panax ginseng	Ginseng	Renshen	Not mentioned
		Curcuma zedoaria	Rhizoma Curcumae	Ezhu	
		Radix salviae miltiorrhizae	Danshen root	Danshen	
		Semen coicis	Coix seed	Yiyiren	
		Spreading hedyotis herb	Hedyotis diffusa	Baihuasheshecao	
		Portulaca grandiflora hook	Scutellariae barbatae	Banzhilian	
		Bletilla striata	Bletilla	Baiji	
		Rehmannia glutinosa	Smilax glabra	Tufuling	
		Semen persicae	Peach seed	Taoren	
		Rafetus swinhoei	Cantharides	Banao	
		Lycium dasystemum Pojark	Babury wolfberry fruit	Gouqizi	
		Trionyx sinensis	Trionycis carapax	Biejia	
		Rhizoma paridis	Flea	Zaoxiu	
		Radix sophorae flavescentis	Lightyellow sophora root	Kushen	

Wen 1996	Jianpi Kangai decoction	Pseudostellaria heterophylla	Radix pseudostellariae	Taizishen	12
		Atractylodes macrocephala koidz	Roasted rhizoma atractylois	Chaobaizhu	12
		Poria	Poria cocos	Fuling	12
		Rhizoma pinelliae ternatae	Pinellia tuber	Banxia	12
		ericarpium citri reticulatae	Tangerine Peel	Chenpi	12
		Radix salviae miltiorrhizae	Danshen root	Danshen	20
		Sargentodoxa cuneata.	Sargent gloryvine	Hongteng	20
		Smilax china L	Smilax china	Baqia	30
		Concha ostreae	Oysters	Shengmuli	30
		Prunella vulgaris	Self heal	Xiakucao	30

Xu 2004	TCM formulae decoction	Radix astragali seu	Milkvetch Root	Shenghuangqi	Not mentioned
		Rehmannia glutinosa Libosch	Radix rehmanniae	Shengdihuang	
		Pseudostellaria heterophylla	Radix pseudostellariae	Taizishen	
		Dioscorea nipponicaMakino	Ningpo yam rhizome	Chuanshanlong	
		Radix salviae miltiorrhizae	Danshen root	Danshen	
		Leonurus artemisia	Radix salviae miltiorrhizae	Yimucao	
		Periostracum cicadae	Cicada Slough	Chantui	
		Achyranthes bidentata blume	Achyranthes longifolia	Huainiuxi	

Yan 1995	TCM formulae decoction	Astragalus mongholicus	Milkvetch root	Huangqi	15
		Codonopsis pilosula	Codonopsis	Dangshen	15
		Atractylodes macrocephala Koidz	Largehead atractylodes rhizo	Baizhu	15
		Poria	Poria cocos	Fuling	12
		Radix Angelicae Sinensis	Chinese angelica	Danggui	15
		Colla corii Asini	colla corii asini	Ajiao	15
		Lycium dasystemum Pojark	Babury Wolfberry Fruit	Gouqizi	15
		Fructus psoraleae	Psoraleae	Buguzhi	15
		Fallopia multiflora	Tuber fleeceflower root	Heshouwu	15
		Spreading hedyotis Herb	Hedyotis diffusa	Baihuasheshecao	20
		Herba cirsii setosi	Common cephalanoplos herb	Xiaoji	15
		Radix salviae miltiorrhizae	Danshen root	Danshen	15
		Millettia dielsiana	Caulis spatholobi	Jixueteng	15
		Sparganium simplex Huds	Rhizoma spargani	Sanleng	15
		Curcuma zedoaria	Rhizoma curcumae	Ezhu	15

Zeng 2015	Fuzhengkangai decoction	Cynanchum paniculatum	Paniculate swallowwort root	Xuchangqing	20
		Atractylodes macrocephala	Largehead atractylodes rhizo	Shengbaizhu	15
		Turmeric Curcumae	Turmeric	Yujin	12
		Radix salviae miltiorrhizae	Danshen root	Danshen	30
		ArtemisiacapillarisThunb	Virgate wormwood herb	Yinchen	30
		Fructus citri aurantii immatur	Immature bitter orange	Chaozhishi	15
		Portulaca grandiflora hook	Scutellariae barbatae	Banzhilian	30
		Solanum lyratum thunb	Solanum lyratum thunb	Baiying	20
		Astragalus mongholicus	Milkvetch root	Shenghuangqi	40
		Cleistocactus sepium	Cuttlebone	Wuzeigu	20
		Curcuma zedoaria	Rhizoma curcumae	Ezhu	10
		Rhizoma Coptidis	Coptis root	Huanglian	6
		Trichosanthes kirilowii Maxim	Pericarpium trichosanthis	Gualoupi	15

Zhang 2013	Xidan Tang	Astragalus mongholicus	Milkvetch root	Huangqi	30
		Bletilla striata	Bletilla	Baiji	12
		Poria	Poria cocos	Fuling	15
		Ganoderma Lucidum Karst	Lucid ganoderma	Lingzhi	15
		Radix salviae miltiorrhizae	Danshen root	Danshen	10
		Rhizoma Ligustici Chuanxiong	Sichuan lovage rhizom	Chuanxiong	9
		Euchresta japonica Hook	Vietnamese sophora root	Shandougen	6
		Fructus Camptothecae Acuminatae	Common Camptotheca Fruit	Xishuguo	10
		Glycyrrhiza uralensis	Liquorice root	Gancao	5

**Table 4:** The top 9 frequency Chinese herb medicines of formulae.

Latin name	English name	Chinese name	Frequentcy	The total frequency (%)	Cumulative percentiles (%)
Radix salviae miltiorrhizae	Danshen root	Danshen	13	100	17.11
Astragalus mongholicus bunge	Milkvetch root	Huangqi	8	61.54	10.53
Rhizoma atractylodis macrocephalae	Largehead atractylodes Poria cocos	Baizhu	5	38.46	6.76
Curcuma zedoaria	Rhizoma curcumae	Ezhu	4	30.77	5.26
Poria	Coastal glehnia root	Fuling	4	30.77	5.26
Agkistrodon seu bungarus	Hedyotis diffusa	Baihuasheshecao	3	23.08	3.95
Adenophora stricta	Radix Adenophorae	Shashen	3	23.08	3.95
Glycyrrhiza uralensis	Liquorice root	Gancao	3	23.08	3.95
Portulaca grandiflora hook	Scutellariae barbatae	Banzhilian	3	23.08	3.95

**Table 5:** Risk of bias assessments for included studies.

Included studies	A	B	C	D	E	F	G	Total
Chen 2012	+	?	+	+	+	?	+	4
Gong 2018	+	?	+	+	+	?	+	5
Guo 2017	+	?	?	+	+	+	+	5
Lan 2003	+	-	+	-	+	?	+	4
Li 2017	+	-	-	?	+	+	+	4
Mok 2007	+	?	+	+	+	+	+	5
Shen 2017	+	?	-	?	+	?	+	4
Wang 2010	+	-	-	?	+	+	+	4
Wen 1996	+	?	?	-	+	+	+	4
Xu 2004	+	?	?	+	+	-	+	4
Yan 1995	+	-	?	+	+	-	+	4
Zeng 2015	+	-	?	?	+	+	+	4
Zhang 2013	+	?	-	?	+	+	+	4

A, random sequence generation (selection bias); B, allocation concealment (selection bias).

C, blinding of participants and personnel (performance bias); D, blinding of outcome assessment(detection bias).

E, incomplete outcome data (attrition bias); F, selective reporting (reporting bias); G, other bias. +, low risk of bias;–, high risk of bias; ?, unclear risk of bias.
